# Active bone marrow dose reconstruction in a large‐scale cohort study of cancer patients treated with photon radiotherapy

**DOI:** 10.1002/acm2.70494

**Published:** 2026-02-24

**Authors:** Keith T. Griffin, Kishan J. Pithadia, Yeon Soo Yeom, Lior Braunstein, Kelly L. Bolton, Lindsay M. Morton, Choonsik Lee

**Affiliations:** ^1^ Division of Cancer Epidemiology and Genetics National Cancer Institute Rockville Maryland USA; ^2^ Department of Radiation Oncology University of Texas Southwestern Medical Center Dallas Texas USA; ^3^ Department of Radiation Convergence Engineering Yonsei University Wonju Gangwon Republic of Korea; ^4^ Department of Radiation Oncology Memorial Sloan Kettering Cancer Center New York New York USA; ^5^ Department of Medicine Washington University School of Medicine St. Louis Missouri USA

**Keywords:** Active bone marrow, computational dosimetry, radiotherapy

## Abstract

**Background:**

Previous studies have investigated the dose‐response relationship between external beam radiotherapy (EBRT) and leukemia by reconstructing the mean active bone marrow (ABM) dose as part of the exposure assessment. However, no prior study has leveraged electronic medical records (EMR) to reconstruct ABM dose and dose‐volume in an EBRT patient population.

**Purpose:**

To support future studies on the relationship between radiation exposure and adverse health effects among EBRT patients, we demonstrate methods to retrospectively calculate ABM dose and dose‐volume metrics in a large patient cohort using EMR.

**Methods:**

We retrieved complete EMR for 639 individuals (five pediatric and 634 adult) from a retrospective cohort of cancer patients treated with photon radiotherapy between 2003 and 2011, covering a wide range of treatment sites and target volumes. Using a previously published methodology by our group, bone site contours were derived for each patient treatment session via three‐dimensional skeletal registration with a habitus‐matched computational phantom. Segmentation accuracy was assessed analytically and by visual inspection of the patient‐phantom skeletal overlap. ABM dose and dose‐volume metrics were computed using the derived skeletal segmentation and the dose distribution calculated by the treatment planning system.

**Results:**

Analytically and visually acceptable skeletal segmentation was achieved for 478 patients (three pediatric, 475 adult). Across all treatment sessions, pelvic irradiation resulted in the highest mean ABM dose per prescribed dose (distribution 25th–75th percentile: 0.1820–0.2870 Gy/Gy_Rx, *n* = 183), while intracranial irradiation produced the lowest among the categorized field types (0.0030–0.0391 Gy/Gy_Rx, *n* = 37). Dose‐volume analyses indicated that the mean ABM dose estimate provides a limited characterization of the ABM dose distribution, with most patients having a 99th percentile dose to marrow volume that was at least one order of magnitude higher than the mean dose estimate.

**Conclusions:**

This study presents a scalable EMR‐based approach for reconstructing ABM dose and dose‐volume metrics in EBRT patients. By capturing clinically relevant dose heterogeneity, we have estimated a more informative exposure variable than mean ABM dose for studies on the role of radiotherapy in leukemogenesis and other adverse health effects. Future work will improve the generalizability of these methods for certain treatment regions and pediatric populations.

## 1 INTRODUCTION

Ionizing radiation exposure is an established risk factor for leukemia.^1^ However, prior reports of the magnitude of the risks associated with high doses from therapeutic radiation have been inconsistent, possibly because of differences in exposure assessment.^2^, ^3^, ^4^, ^5^, ^6^, ^7^, ^8^ The target cells responsible for leukemogenesis are considered to be the hematopoietic stem cells of the active bone marrow (ABM),^9^ a distributed tissue spread out amongst skeletal sites throughout the body in an age‐ and sex‐dependent manner.^10^ Most prior studies of subsequent leukemia with dose reconstruction have evaluated the mean ABM dose estimated across several compartments of the skeleton^11^ or across a multitude of point locations throughout the skeleton.^12^ However, prior studies have not considered the proportion of marrow exposed to the treatment field, or the dose volume.^3^ As dosimetry techniques evolve, new methods for deriving dose‐volume may improve the accuracy by which we assess radiation exposure and enhance our understanding of its relationship with cancer risks.^13^ This may be particularly true for distributed, whole‐body tissues such as the ABM that experience a large dose gradient from most forms of external beam radiotherapy.

Two prior studies have demonstrated methods for retrospectively calculating dose‐volume metrics in the ABM of patients treated with external beam radiotherapy. One study by Veres et al. estimated the ABM dose and dose‐volume on a surrogate whole‐body patient anatomy using analytical calculations based on measurements in a water phantom,^14^ a method that has been applied for patient data from time periods prior to the widespread adoption of electronic medical records.^15^, ^16^, ^17^ Another method capable of producing dose‐volume metrics was recently published by our group, demonstrating a novel process to calculate ABM dose and dose‐volume using collected electronic medical record data. This method automatically contours bone sites on the patient computed tomography (CT) image set by matching the skeleton of the patient to that of a whole‐body computational human phantom with detailed pre‐segmented bone sites; dose estimates then come directly from the dose distribution calculated by the hospital treatment planning system.^18^

In the current study, we sought to apply our previously published methods to a subset of cancer patients within the Memorial Sloan Kettering‐Integrated Mutation Profiling of Actionable Cancer Targets (MSK‐IMPACT) cohort. A recent study within the MSK‐IMPACT cohort has shown a significant association between treatment with external beam radiotherapy and the development of clonal hematopoiesis,^19^ a known precursor to leukemia^20^, ^21^, ^22^; however, ABM dose estimates were not calculated in the initial analyses of that study. To this end, we aimed to support future analyses on the relationship between radiation exposure and clonal hematopoiesis within the MSK‐IMPACT study by estimating the ABM dose and dose‐volume to cohort members for whom electronic medical records were available. Our improved exposure assessment methodology can serve to inform future radiotherapy planning strategies by illuminating the relative influence of ABM mean dose and dose‐volume on adverse effects. To our knowledge, this is the first attempt to retrospectively calculate ABM dose and dose‐volume in a large cohort of radiotherapy patients using electronic medical records, providing an individualized radiation exposure assessment for the ABM in each patient.

## 2 METHODS

### 2.1 Radiotherapy data collection

A total of 850 patients were selected for dose reconstruction. Inclusion criteria required that patients received all primary cancer treatment at Memorial Sloan Kettering, were treated with external beam radiotherapy, and had electronic medical records available within the MSK‐IMPACT cohort. The collected electronic data were stored in the Digital Imaging and Communications in Medicine for Radiation Treatment (DICOM RT) format. This datatype encompasses a series of files, including: the computed tomography (CT) image set of the patient, the segmentation structure file (RT‐struct), the radiotherapy treatment plan file (RT‐plan), and the radiotherapy dose distribution file (RT‐dose). Important patient characteristics and treatment information were extracted from the cohort database, including age, sex, height, and weight at the time of treatment, tumor treatment site, and total delivered dose. The delivered dose variable was used in an initial quality assurance check on the completeness of the electronic records. Scripts were written to extract the prescribed dose per radiation beam from the DICOM RT‐plan file, the sum of which was compared against the total delivered dose for each patient. In the current analysis, 178 patients with mismatching dose greater than 0.1 Gy between the two sources of information were excluded from the current study. Radiation was delivered using megavoltage photon machines at a nominal energy between 6 and 20 MV. Patient treatment fields were categorized based on the RT‐plan label and tumor treatment site (when available from the database), as well as a visual evaluation of the treatment isocenter on the CT; further description on treatment field categorization by region of the body may be found in the Appendix.

### 2.2 Patient active bone marrow dose reconstruction

#### 2.2.1 Phantom‐based skeletal segmentation

A workflow previously developed by our group^18^ was adopted in the current study to provide segmentation of the skeleton of the patient within the virtual coordinate system of the DICOM RT data. Our tool provides the capability to automatically contour the individual bone sites of the patient by matching the whole skeleton of the patient to that of a computational human phantom via three‐dimensional registration. The computational phantom used for matching is pulled from a library of body‐size dependent phantoms developed by the University of Florida and National Cancer Institute (UF/NCI).^23^ Three hundred and fifty‐one phantoms are available in this library, differentiated by age, sex, height, and weight; from highest to lowest priority, respectively, these patient characteristics are used to select the closest matching phantom for the alignment process. The skeleton of the whole‐body computational phantom comes pre‐contoured, categorized into 34 bone sites and containing separately defined regions of cortical bone, trabecular spongiosa, and medullary cavity (of the long bones). The skeleton of the patient, which is derived from CT Hounsfield unit thresholding, and the skeleton of the whole‐body computational phantom are registered by performing the iterative closest point algorithm.^24^ Once complete, the positions of each voxel representing a bone site within the phantom have been derived within the virtual coordinate system of the original DICOM series, allowing the bone site contours within the phantom to overlay the patient dose distribution within the RT‐Dose file.

#### 2.2.2 Skeletal segmentation quality assurance

Application of our automated methodology to a substantially larger number of patients than in our earlier study^18^ warranted quality assurance measures to ensure a proper patient‐to‐phantom skeletal alignment has been performed for all patients. In the previous study, the volume overlap fraction (VOF) was calculated to compare the similarity between the patient skeletal and phantom skeletal position after alignment:

(1) $$VOF=\frac{\left|S_{patient}\cap S_{phantom}\right|}{\left|S_{patient}\right|}\;\;x\;100\;\left(\%\right)$$

where $S_{patient}$ and $S_{phantom}$ are the set of voxels associated with the skeleton of the patient and phantom, respectively. A higher VOF from Equation (1) represents a better overlap between the two anatomies. However, a poor VOF score does not necessarily indicate poor accuracy in the ABM dose estimate. Geometric agreement tends to be worse for more complex and thinner bones, such as the ribs in abdominal and thoracic treatments, though the distance between the patient and phantom bone site is typically small. Additionally, the dose error can be small despite low VOF for bones that lie within the treatment field, where the dose distribution is nearly uniform, and for bones that lie far from the treatment field, where absolute doses are small. For these reasons, VOF was only used in the current study as a comparative metric against the VOF scores validated in our prior study containing manual skeletal segmentations for 40 patients in the cohort.^18^

**FIGURE 1:**
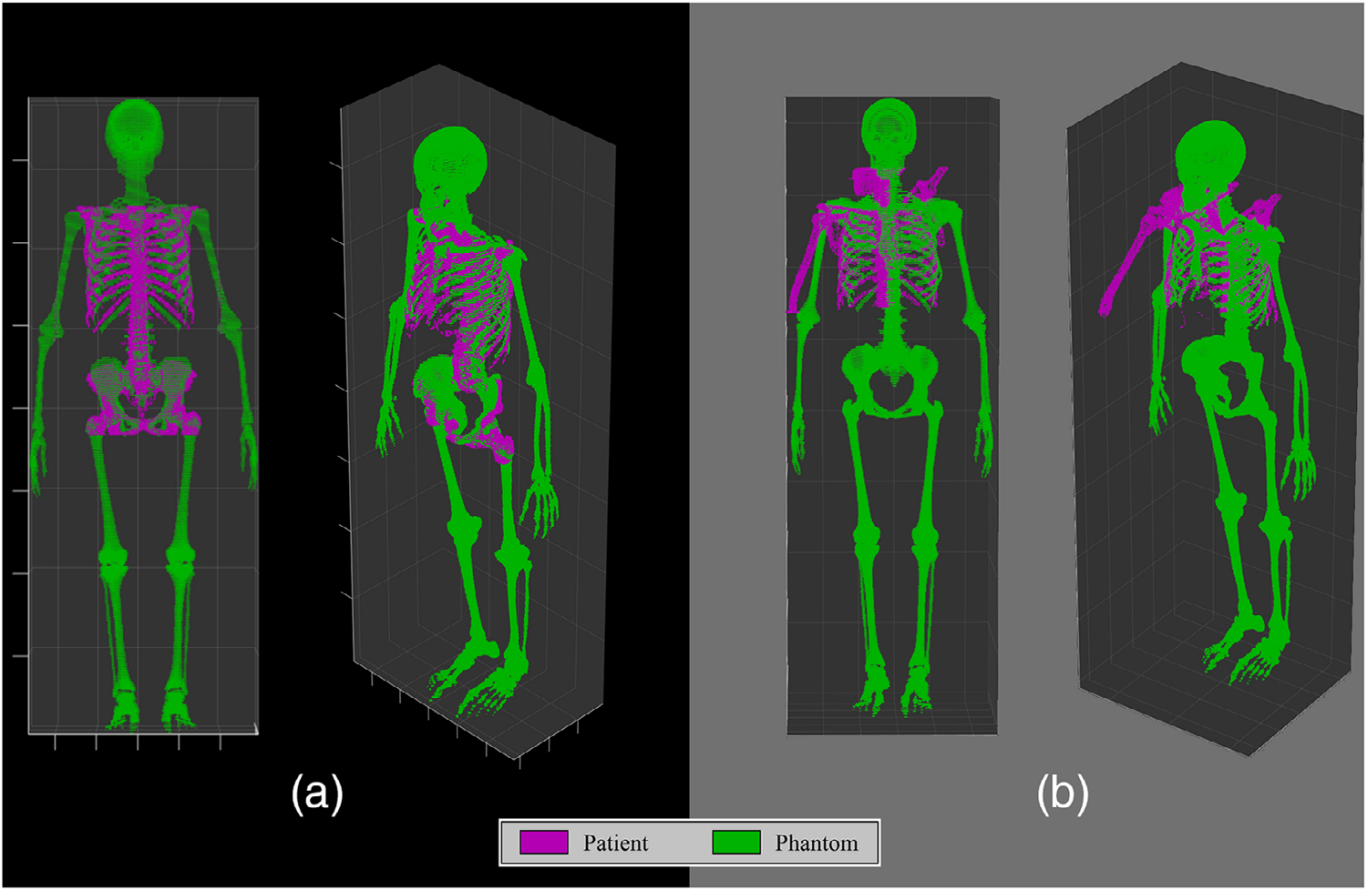
Visualization of the patient‐phantom skeletal alignment for two example patients. Each colored point represents one skeletal voxel in either the patient (purple) or phantom (green). (A) 75th percentile patient‐phantom alignment score (VOF of 41.1%) that was accepted; (B) 25th percentile patient‐phantom alignment score (VOF of 21.4%) that was rejected.

Skeletal registrations were instead manually inspected by two independent evaluators. For this process, images were written out following registration that depict the three‐dimensional skeletal point sets for both the patient and the phantom. Example images are depicted in Figure 1, where the purple set of points are the skeletal positions in the patient and the green set of points are those in the matched computational phantom. Registration images that showed excellent overlap between the two skeletal point sets, particularly in the region being treated, were accepted if both evaluators agreed; the left side of Figure 1 shows an example accepted alignment for a spinal irradiation treatment. Registrations identified as having poor alignment were excluded from the final analytical study population if one or more evaluators rejected the segmentation; the right side of Figure 1 shows an example rejected alignment for a breast irradiation treatment. Potential biases of the skeletal segmentation method with respect to patient characteristics (i.e., age, sex, height, and weight) were identified through calculation of the Spearman's rank‐order correlation coefficient; this metric was chosen based on its robustness to skewed distributions and ability to work with binary data. Associations were explored between these factors and the VOF score within the set of accepted segmentations.

#### 2.2.3 Active bone marrow dose and dose‐volume reconstruction from skeletal contours

Positions of the contoured bone sites within the virtual coordinate system of the DICOM series were used to assign dose from the RT‐dose file to each skeletal voxel; zero dose was assigned to the skeletal voxels outside the boundaries of the dose distribution matrix. Patient ABM dose was then calculated by assigning a fraction of the total active marrow to each skeletal voxel within the patient‐matched phantom, excluding cortical bone. ABM fractions were sourced from a derivation of age‐ and sex‐specific marrow fractions at each bone site recently published by the International Commission on Radiological Protection.^25^ These fractions of total ABM, as shown in Table 1, were homogeneously distributed to the set of trabecular bone or medullary cavity voxels at each bone site. Mean dose to the ABM was calculated as the average dose across all bone voxels weighted by their marrow fractions, and dose‐volume histograms were calculated using these same weights in the contribution to the overall dose distribution. Additionally, dose metrics, such as V# (the percentage of ABM volume receiving at least # Gy) and D# (the percentile dose in the ABM, e.g., D10 is the 90^th^ percentile dose) were computed. The entirety of the segmentation and dosimetry workflow was written and executed in MATLAB script (R2023a) on the National Institutes of Health High Performance Supercomputing Cluster (hpc.nih.gov) taking less than 2 min per patient using a 2.6 GHz Intel E5‐2650v2 processor.

**TABLE 1:** Active bone marrow distribution as a function of age and sex.

	Fraction of total active bone marrow by age (year) and sex (F: female; M: male)							
Bone site	00F/M	01F/M	05F/M	10F/M	15F	15 M	30F	30 M
Craniofacial bones	0.2412	0.3274	0.3254	0.1753	0.0983	0.1198	0.0318	0.0447
Mandible	0.0433	0.0167	0.0212	0.0108	0.0078	0.0099	0.0086	0.0082
Scapulae	0.0323	0.0339	0.0404	0.0448	0.0380	0.0277	0.0742	0.0834
Clavicles	0.0122	0.0044	0.0080	0.0092	0.0085	0.0066	0.0097	0.0098
Sternum	0.0051	0.0054	0.0113	0.0162	0.0149	0.0214	0.0204	0.0240
Ribs	0.1628	0.1506	0.0850	0.0948	0.1036	0.0951	0.1244	0.0937
Cervical vertebrae	0.0373	0.0270	0.0152	0.0202	0.0316	0.0252	0.0327	0.0295
Thoracic vertebrae	0.0516	0.0944	0.0955	0.1525	0.1132	0.1252	0.1245	0.1199
Lumbar vertebrae	0.0470	0.0524	0.0640	0.1035	0.1385	0.1261	0.1559	0.1661
Sacrum	0.0188	0.0359	0.0430	0.0399	0.0684	0.0559	0.0651	0.0744
Os coxae	0.0631	0.1007	0.1138	0.1812	0.2460	0.2205	0.2495	0.2442
Femora, proximal head	0.0346	0.0164	0.0216	0.0342	0.0593	0.0772	0.0491	0.0415
Femora, upper shaft	0.0103	0.0048	0.0138	0.0152	0.0109	0.0150	0.0109	0.0258
Femora, lower shaft	0.0164	0.0074	0.0095	0.0100	0.0071	0.0066	0.0	0.0
Femora, distal head	0.0286	0.0170	0.0235	0.0183	0.0	0.0	0.0	0.0
Tibiae, proximal head	0.0246	0.0099	0.0121	0.0089	0.0	0.0	0.0	0.0
Tibiae, shaft	0.0094	0.0102	0.0109	0.0077	0.0	0.0	0.0	0.0
Tibiae, distal head	0.0150	0.0021	0.0038	0.0022	0.0	0.0	0.0	0.0
Fibulae, proximal head	0.0033	0.0005	0.0012	0.0007	0.0	0.0	0.0	0.0
Fibulae, shaft	0.0015	0.0010	0.0025	0.0016	0.0	0.0	0.0	0.0
Fibulae, distal head	0.0050	0.0004	0.0011	0.0007	0.0	0.0	0.0	0.0
Patellae	0.0013	0.0016	0.0059	0.0033	0.0	0.0	0.0	0.0
Ankles and feet	0.0375	0.0254	0.0176	0.0	0.0	0.0	0.0	0.0
Humeri, proximal head	0.0212	0.0148	0.0166	0.0245	0.0469	0.0589	0.0394	0.0283
Humeri, upper shaft	0.0045	0.0041	0.0063	0.0059	0.0047	0.0059	0.0039	0.0065
Humeri, lower shaft	0.0045	0.0038	0.0058	0.0058	0.0023	0.0030	0.0	0.0
Humeri, distal head	0.0166	0.0068	0.0083	0.0055	0.0	0.0	0.0	0.0
Radii, proximal head	0.0036	0.0005	0.0008	0.0004	0.0	0.0	0.0	0.0
Radii, shaft	0.0017	0.0017	0.0031	0.0018	0.0	0.0	0.0	0.0
Radii, distal head	0.0065	0.0010	0.0016	0.0009	0.0	0.0	0.0	0.0
Ulnae, proximal head	0.0086	0.0019	0.0030	0.0017	0.0	0.0	0.0	0.0
Ulnae, shaft	0.0020	0.0028	0.0035	0.0020	0.0	0.0	0.0	0.0
Ulnae, distal head	0.0044	0.0001	0.0004	0.0002	0.0	0.0	0.0	0.0
Wrists and hands	0.0242	0.0171	0.0043	0.0	0.0	0.0	0.0	0.0

## 3 RESULTS

### 3.1 Skeletal alignment performance and final analytical study population

**FIGURE 2:**
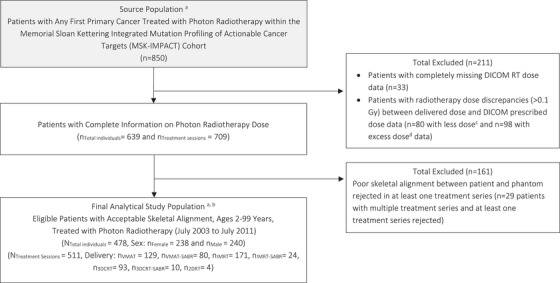
Flowchart of study population selection from the MSK‐IMPACT cohort. Digital imaging and communications in medicine for radiation treatment (DICOM RT), volumetric modulated arc therapy (VMAT), stereotactic ablative radiotherapy (SABR), intensity modulated radiotherapy (IMRT), three‐dimensional conformal radiotherapy (3DCRT), two‐dimensional radiotherapy (2DRT), electronic medical records (EMR). *Notes*: ^a^ All cases included are linked to EMR; ^b^ In the current work, certain analyses were conducted at the level of individual treatment sessions, with some patients undergoing more than one session. Among the 478 patients selected, 32 had multiple treatment sessions, totaling 511 sessions across the population; ^c^ Indicative of one or more missing DICOM files; ^d^ Suggestive of one or more undelivered DICOM sessions, leading to uncertainty on which were delivered.

Among the 850 patients treated with photon radiotherapy in our sub‐study of the MSK‐IMPACT cohort, 478 patients with quality dosimetry have been selected for our final analytical study population. Figure 2 depicts this selection flowchart. There were 211 patients who were excluded due to a mismatch between the records of delivered dose from the cohort database and the dose information held within the DICOM RT‐plan and RT‐dose files: 33 patients with completely missing DICOM RT‐dose data, 80 patients with lower prescribed dose in the RT‐plan file than recorded in the database, and 98 patients with higher prescribed dose in the RT‐plan file than recorded in the database. Among the 639 remaining patients that had complete photon radiotherapy dose available, 60 had multiple treatment sessions, summing to a total of 709 individual treatment sessions evaluated with the skeletal alignment methodology. The distribution of these 709 treatment sessions categorized by treatment region and skeletal alignment performance can be seen in Table 2. In total, the skeletal alignment methodology provided acceptable segmentations for 542 of the 709 treatment sessions, leading to an overall acceptance rate of 76.4%. The most accurate skeletal alignments were seen in treatment sessions involving an intracranial target volume, with an acceptance rate of 95.7%; the least accurate alignments were seen in target volumes within the extremities, where only one treatment session out of twenty‐one was accepted. From these 542 sessions, only 511 were ultimately selected, as some patients with multiple delivered treatments were rejected if any of their individual sessions were not accepted. Thus, a total of 161 patients were excluded due to a combined rejection of 198 treatment sessions. Within the accepted treatment sessions, the median VOF score was 34.1%; these scores were similar to the results of our prior pilot study, where mean bone‐specific VOF between 4% (clavicles) and 52% (pelvis) resulted in a mean ABM dose error of 0.4 Gy.^18^ Descriptions of the VOF distribution of the accepted treatment sessions and their comparison to the validated results of our prior study may be found in the Appendix. Within the set of adult patients, statistical analyses showed significant but weak associations between skeletal alignment performance (i.e., VOF) and patient age, sex, height, and weight, demonstrating the generalizability of the model to a variety of adult patient body characteristics; further descriptions of this analysis may also be found in the Appendix. The sample size of pediatric treatment sessions was insufficient to judge the performance of our methods for younger patient ages.

**TABLE 2:** Overview of radiotherapy treatment sessions and selection based on skeletal alignment.

		Selection of treatment sessions based on skeletal alignment *n* (%) ^c^		
Treatment region ^a^	Distribution of total available treatment sessions *n* (%) ^b^	Accepted	Rejected	Distribution of accepted treatment sessions *n* (%) ^b^, ^d^
**Overall sample**	709 (100%)	542 (76.4%)	167 (23.6%)	511 (100%)
**Abdominal**	75 (10.6%)	47 (62.7%)	28 (37.3%)	46 (9.0%)
**Bone**	42 (5.9%)	30 (71.4%)	12 (28.6%)	28 (5.5%)
***Extremities***	*12 (1.7%)*	*1 (8.3%)*	*11 (91.7%)*	*0 (‐)*
***Pelvic***	*24 (3.4%)*	*24 (100%)*	*0 (‐)*	*24 (4.7%)*
***Thoracic***	*6 (0.8%)*	*5 (83.3%)*	*1 (16.7%)*	*4 (0.8%)*
**Breast**	74 (10.4%)	49 (66.2%)	25 (33.8%)	47 (9.2%)
**Central nervous system**	116 (16.4%)	95 (81.9%)	21 (18.1%)	75 (14.7%)
***Intracranial***	*47 (6.6%)*	*45 (95.7%)*	*2 (4.3%)*	*37 (7.2%)*
***Spine***	*69 (9.7%)*	*50 (72.5%)*	*19 (27.5%)*	*38 (7.5%)*
**Extremities**	9 (1.3%)	0 (‐)	9 (100%)	0 (‐)
**Head and neck**	57 (8.0%)	39 (68.4%)	18 (31.6%)	37 (7.2%)
**Pelvic**	217 (30.6%)	185 (85.3%)	32 (14.7%)	183 (35.8%)
**Thoracic**	119 (16.8%)	97 (81.5%)	22 (18.5%)	95 (18.6%)

*Notes*: The current analysis is based on treatment sessions for all patients with complete information on the photon radiotherapy dose, with some individuals having more than one session. Of these 639 patients, 60 had multiple treatment sessions, summing to a total of 709 sessions evaluated with the skeletal alignment methodology.

^a^ Treatment region categorization is specified within the Appendix.

^b^ Percentages (column) indicate the distribution of treatment sessions across treatment regions.

^c^ Percentages (row) represent the proportion of sessions accepted and rejected for each treatment region.

^d^ Thirty‐one treatment sessions were ultimately rejected that had acceptable skeletal alignment but were delivered to a patient who was excluded due to a separate rejected treatment session.

Within the 511 accepted treatment sessions, the most commonly treated area was the pelvic region (35.8%), followed by the thoracic region (18.6%), and then the central nervous system (14.7%). The least common was within thoracic bones (0.8%), and no treatment sessions involving the extremities were selected in the final sample. The final analytical study population is comprised of 478 patients, 238 males and 240 females, treated between July 2003 and July 2011. Of these patients, 447 had one treatment session, 29 had two treatment sessions, and 2 had three treatment sessions. The delivery technique varied across these sessions, with the largest proportion categorized as volumetric modulated arc therapy (41%), followed by intensity modulated radiotherapy (38%), three‐dimensional conformal radiotherapy (20%), and two‐dimensional radiotherapy (<1%). Roughly one‐fifth were delivered with treatment characteristics typical of stereotactic ablative radiotherapy (e.g., a delivery of large target dose in a few fractions). The median patient age at the time of treatment was 63.6 years with an interquartile range from 52.7 to 70.7 years. Only three patients were under the age of 21.

### 3.2 Active bone marrow dose and dose‐volume estimates

**FIGURE 3:**
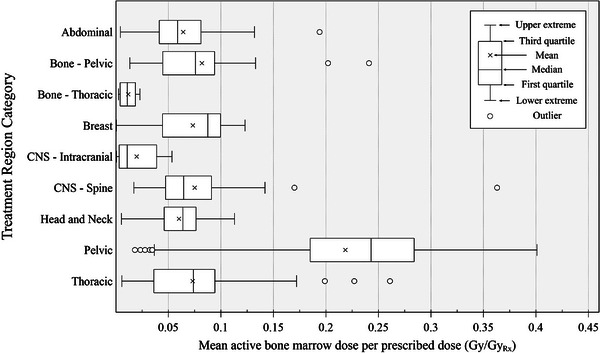
The distribution of mean active bone marrow dose per prescribed dose across all individual radiotherapy treatment sessions in the final study population, categorized by the region of the body being treated. The current analysis is based on 511 treatment sessions from 478 patients accepted in our final study population. Whiskers and outliers were determined using the 1.5 interquartile range rule. Central nervous system (CNS).

The range of ABM doses by treatment region amongst the final analytical study population is indicated by the length of the boxplots in Figure 3. From these results, we have identified a correlation between the treated region of the body and ABM dose‐volume metrics. To a substantial degree, treatments involving the pelvic region were associated with the highest mean ABM doses, partly due to the high volume of active marrow contained in the pelvis, sacrum, lumbar vertebrae, and femoral heads for an adult population. Conversely, intracranial fields delivered the lowest mean doses. The mean ABM dose per prescribed dose showed the following descending order by treatment region: pelvic (median across the study population: 0.2430 Gy/Gy_Rx), breast (0.0875), pelvic bone (0.0758), thoracic (0.0738), spine (0.0645), head and neck (0.0638), abdominal (0.0587), thoracic bone (0.0109), and intracranial (0.0108) regions. Similarly, the highest mean ABM dose recorded in a single treatment session was delivered to the pelvic region (18.8 Gy), while the lowest was in an intracranial treatment (0.017 Gy). Relatively low mean dose estimates were also seen for fields involving thoracic and pelvic bones, even though the thoracic and pelvic bone sites of the adult body hold a relatively high fraction of the active marrow. This trend was due to the smaller target volumes from treatments to bone compared with other treatments in this study.

Dose‐volume metrics across all treatment sessions in this study population are summarized in Table 3. On average, treatments to the pelvic region delivered the highest median ABM dose at 6.97 Gy. On the other hand, treatments to certain regions of the body, including thoracic bone, breast, intracranial, and head and neck regions, were estimated to deliver zero median dose, indicating that more than half of the ABM within the patient was estimated to have received no dose. Across most treated regions of the body, the metric for near‐maximum dose to the ABM, D1, was roughly equivalent to the prescribed dose, demonstrating that some portion of the active marrow was typically within or nearby the treatment volume.

**TABLE 3:** Active bone marrow dose‐volume metrics across all radiotherapy treatment sessions in the final study population.

		Averaged dose‐volume metrics ^b^ [Q1, Q3]					
Treatment region ^a^	Averaged Rx. dose (Gy)	D50 (Gy)	D10 (Gy)	D1 (Gy)	V5 (%)	V10 (%)	V20 (%)
**Abdominal**	44.7	0.008 *[0.0, 0.0]*	4.22 *[1.1, 5.7]*	34.4 *[25.3, 45.6]*	8.3 *[6.1, 10.5]*	6.7 *[5.0, 8.9]*	4.5 *[2.8, 6.5]*
**Bone**	24.8	0.140 *[0.0, 0.18]*	9.48 *[1.5, 18.2]*	22.0 *[19.1, 28.1]*	10.5 *[6.1, 14.9]*	7.9 *[1.8, 10.9]*	5.3 *[0.93, 9.6]*
***Pelvic***	24.6	0.163 *[0.17, 0.25]*	11.0 *[3.0, 19.3]*	23.6 *[20.8, 28.1]*	12.1 *[8.1, 15.9]*	9.1 *[5.2, 12.2]*	6.1 *[1.9, 9.6]*
***Thoracic***	26.0	0.0 *[0.0, 0.0]*	0.158 *[0.06, 0.19]*	12.3 *[1.4, 20.2]*	1.2 *[0.23, 1.9]*	0.9 *[0.18, 1.6]*	0.5 *[0.03, 0.9]*
**Breast**	46.9	0.0 *[0.0, 0.0]*	4.17 *[0.4, 7.4]*	45.9 *[43.3, 52.8]*	8.5 *[3.2, 13.4]*	5.7 *[2.8, 8.3]*	4.0 *[2.4, 5.3]*
**Central Nervous System**	30.7	0.085 *[0.0, 0.0]*	2.64 *[0.01, 1.4]*	23.7 *[12.0, 32.0]*	5.6 *[1.8, 6.6]*	4.7 *[1.3, 6.0]*	3.4 *[0.60, 4.9]*
***Intracranial***	32.2	0.0 *[0.0, 0.0]*	1.05 *[0.0, 0.19]*	19.4 *[5.4, 32.3]*	3.9 *[1.1, 5.8]*	3.4 *[0.59, 5.5]*	2.9 *[0.11, 5.3]*
***Spine***	29.2	0.168 *[0.0, 0.09]*	4.20 *[0.43, 2.0]*	27.9 *[22.3, 30.7]*	7.2 *[3.6, 8.0]*	5.8 *[3.2, 7.0]*	3.9 *[1.6, 4.6]*
**Head and Neck**	46.4	0.0 *[0.0, 0.0]*	2.52 *[0.88, 2.9]*	42.1 *[31.0, 56.6]*	6.7 *[4.5, 8.6]*	5.2 *[3.8, 7.0]*	3.7 *[2.4, 5.6]*
**Pelvic**	45.1	6.97 *[1.36, 11.9]*	37.3 *[30.6, 46.8]*	49.1 *[47.0, 53.9]*	43.2 *[38.9, 54.0]*	39.7 *[32.9, 51.2]*	32.3 *[24.9, 42.8]*
**Thoracic**	48.0	0.001 *[0.0, 0.0]*	6.23 *[1.1, 9.1]*	36.6 *[24.5, 50.6]*	9.2 *[5.7, 12.6]*	7.0 *[3.9, 9.6]*	4.7 *[1.8, 7.1]*

*Notes*: The current analysis is based on 511 treatment sessions from 478 individual patients accepted in our final study population.

Abbreviation: Prescription (Rx.), First quartile (Q1), Third quartile (Q3).

^a^ Treatment region categorization is specified within the Appendix.

^b^ Distribution of dose in the active bone marrow volume is defined by D50 (50^th^ percentile dose = median), D10 (90^th^ percentile dose), and D1 (99^th^ percentile dose). V5, V10, and V20 represent the percentage of active bone marrow volume receiving at least 5 Gy, 10 Gy, and 20 Gy, respectively. First and third quartile dose‐volume metrics are presented in brackets.

**FIGURE 4:**
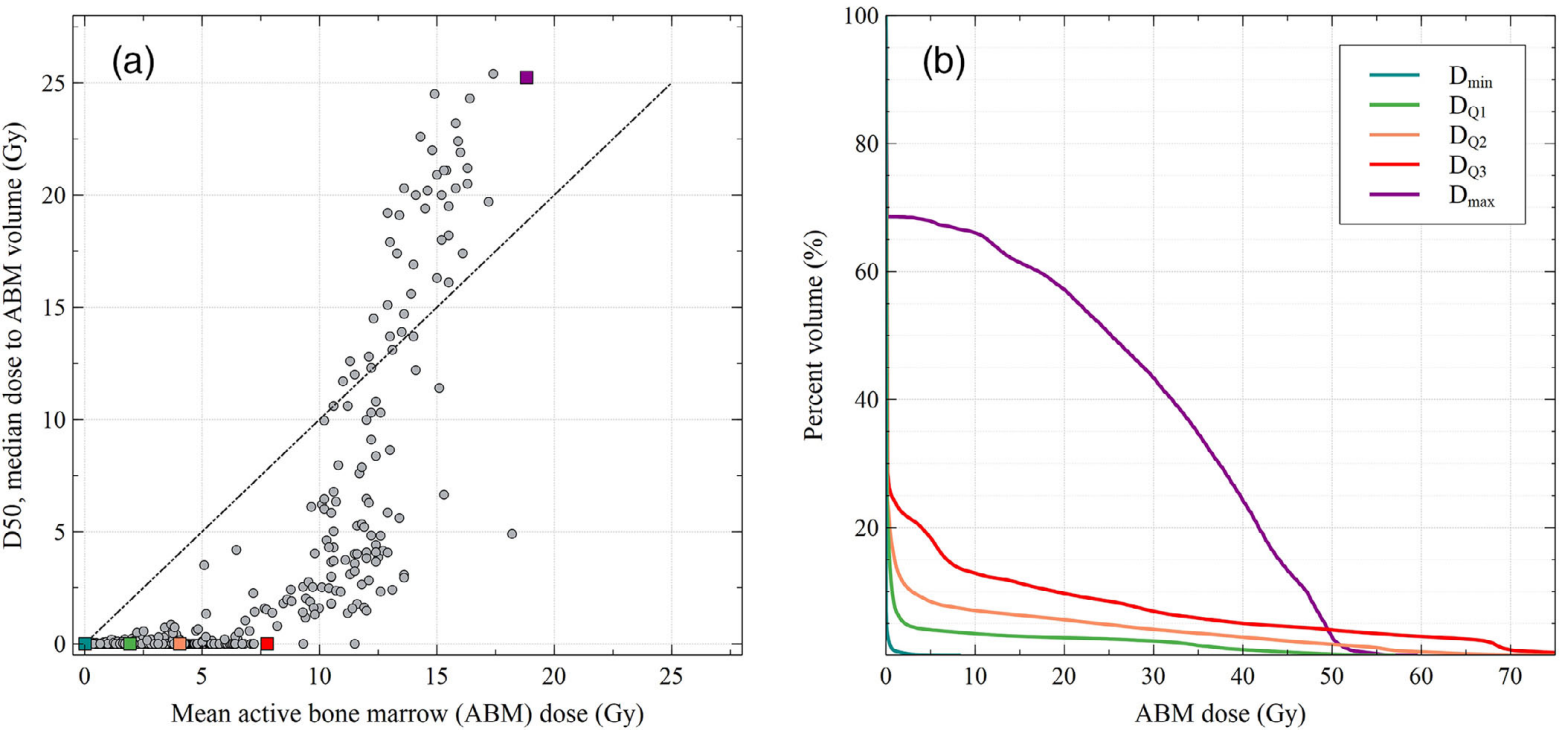
Characteristics of the active bone marrow (ABM) dose‐volume across the final study population. (A) Scatter plot of the median dose to ABM volume (D50) versus mean ABM dose for all 478 patients; the y = x line is plotted for reference. Five highlighted points correspond to the five patients in panel B. (B) Dose‐volume histograms for five example patients, representing the range in mean ABM dose observed in the study: the minimum (D_min—_one intracranial treatment), first, second, and third quartile (D_Q1_ ‐ one thoracic treatment; D_Q2_ – one head and neck treatment; D_Q3_ – two thoracic treatments), and maximum (D_max—_one pelvic treatment).

Panel A of Figure 4 shows the distribution of the ABM dose‐volume metric D50 as a function of mean ABM dose; each point of the scatter plot represents the dose and dose‐volume estimates for one patient within the final study population. These data depict the final dose and dose‐volume estimates for a given patient after the summation of all treatment sessions. An equal D50 metric and mean dose estimate would fall along the y equals x line (plotted as dashed black line) and indicate a dose‐volume distribution symmetric about the median dose to ABM volume. As seen in Figure 4A, the dose‐volume distribution was asymmetric for the majority of patients. At relatively lower mean ABM doses (around 12 Gy or less), the dose‐volume distribution was skewed towards a majority of dose being received by a small volume of ABM (i.e., a mean dose greater than the median dose). At higher mean ABM doses, the opposite tended to be true. Panel B of Figure 4 depicts these distributions as dose‐volume histograms for five example patients, representing the full range in mean ABM dose across the final study population. These five example patients of panel B are also indicated on panel A by their corresponding color. The example dose‐volume histograms seen in Figure 4 for the first, second, and third quartile dose‐volume histograms are typical for most patients in our study: the majority of the ABM was estimated to receive little to no dose while a small portion was estimated to receive dose at or above the prescribed dose. Treatments to the intracranial and thoracic bone regions were an outlier to this trend, as some treatment sessions at these sites utilized stereotactic ablative radiotherapy techniques that led to greatly lower D1 metrics compared to conventional techniques. Treatments to the pelvic region were also an outlier, as seen within Table 3 and in the maximum dose example in Figure 4, as a large proportion of the ABM, on average nearly 40%, received upwards of 10 Gy.

## 4 DISCUSSION

To support future dose and dose‐volume analyses in the MSK‐IMPACT study, we have reconstructed the inadvertent radiation dose to the ABM of 478 patients who were administered photon external beam radiotherapy between 2003 and 2011 for a wide range of cancers (Figure 2, Table 2). Overall, a wide variation in ABM dose per prescribed dose was seen within each categorized treatment region (Figure 3), indicating the importance of patient‐specific dose reconstruction methods. Mean ABM dose estimates were highest in patients undergoing treatments to the pelvic region, on average roughly 9.9 Gy, and lowest in patients undergoing intracranial treatments, on average roughly 0.6 Gy. Compared with previous studies on photon radiotherapy in an adult patient population, the magnitude of the mean ABM estimates of the current work were consistent with or slightly lower than those seen in treatments of breast^2^ (7.5 Gy), uterine corpus^3^ (9.7 Gy), and testicular^4^ cancer (10.9 Gy) from the mid‐ to late‐1900′s.

One meaningful advantage of our approach comes from its capability to provide dose‐volume metrics for the ABM, which we have shown to be more descriptive of the dose heterogeneity experienced by a whole‐body organ as compared to the mean dose (Figure 4, Table 3). Several previous studies on the dose‐response relationship of leukemia risk following radiotherapy^2^, ^3^, ^4^, ^5^, ^6^, ^7^, ^26^ calculated dose using methodology based on physical phantom measurements and mathematical phantom simulations^11^, ^12^; risk estimates were made using either the mean ABM dose or mean dose estimates at compartments of the skeleton. Prior to our recently published method,^18^ only one previous study by Veres et al.^14^ provided methods to retrospectively compute dose‐volume metrics for the ABM in radiotherapy patients; the methods from their study were geared towards historical patient data lacking electronic medical records. In our current work, direct use of the patient anatomy (CT image set) and dose calculated from the treatment planning system (RT‐dose file) allows for a more patient‐specific estimate of the ABM dose, and the availability of dose‐volume metrics affords our future analyses a rare opportunity to gain insight into the dose‐volume effect of cancer initiation.^13^ These analyses may also provide clarification on the relative impact of different dose and dose‐volume metrics on adverse health effects, which can inform new radiotherapy planning strategies regarding ABM dose and dose‐volume. To our knowledge, our efforts represent the first attempt to calculate dose and dose‐volume metrics for the ABM in a large‐scale epidemiological study using electronic medical records.

Although electronic medical records provide a validated source for computing patient‐specific dose, there are limitations to its use. The first limitation is that the dose from the treatment planning system is only calculated on the patient CT image set; thus, the dose estimates to all areas outside of the CT are unknown and in this study were assumed to be zero. Consequently, this assumption introduces a systematic downward bias in the ABM dose estimates, the magnitude of which is dependent on both the proportion of the active marrow outside the CT and the proximity of the treatment volume to the cutoff of the CT image set. Prior work has demonstrated the ability to extend partial‐body CT anatomy with that of another patient,^27^ which may allow for dose extrapolation in future efforts with this cohort. A second limitation is that the accuracy of dose estimates from the treatment planning system is typically limited to the region surrounding the target volume. Several papers have previously acknowledged that the treatment planning system underestimates out‐of‐field dose, particularly as the distance from the field edge grows.^28^, ^29^, ^30^ One advantage of the methods of Veres et al. is in their use of an analytic method to calculate whole‐body ABM dose, which can estimate dose far from the treatment field edge.^14^ A hybrid approach combining dose within and nearby the treatment volume from the treatment planning system with analytical dose calculations further out of field could provide a more accurate estimation of the ABM dose distribution. Note for both limitations, however, that improved characterization of the dose to the portion of ABM far from the treatment field may be inconsequential. This is because these doses are magnitudes lower, and previous studies have indicated the greater impact of in‐field and near‐field tissue doses on adverse health effects.^13^, ^31^

Our phantom‐based automatic segmentation tool has filled a critical research gap, allowing ABM dose to be derived from electronic medical records without the need for manual segmentation. Testing and comparison with the results of our previous study have shown its ability to perform well for a wide range of adult patient body sizes and modern radiotherapy techniques. However, there are some limitations to the applicability of this method. The small number of patients at younger ages within this study limits the generalizability of our tool to pediatric radiotherapy. Future work using pediatric treatment sessions from another cohort would be necessary to test the performance of these methods on younger‐aged patients. Additionally, a modest proportion of patient treatment sessions were unable to be included in this study due to incompatibilities between the patient and phantom anatomies. Our skeletal alignment process is currently limited by the inherently rigid and standardized position of the phantoms, which cannot capture the different patient postures seen across different treatment types. The right side of Figure 1 exhibits one example of a patient treated for breast cancer with a posture that has no match within our computational phantom library. The results from Table 2 have indicated that patients treated at certain sites, such as the extremities, breast, and abdominal regions, would benefit from a technique that provides a more flexible method for skeletal segmentation. One auto‐segmentation tool, TotalSegmentator,^32^ has found increased use in dosimetry studies,^33^, ^34^, ^35^ and a recent update has made TotalSegmentator capable of automatically segmenting all of the individual marrow‐containing bone sites necessary to derive an ABM dose distribution. Future work from our group will attempt to develop new methods to incorporate the rapidly developing field of deep‐learning based auto‐segmentation to recapture the 161 patients that were lost in this study due to poor segmentation. Technical challenges anticipated for these efforts include: the estimation of bone volume outside of the patient CT image, quality assurance of the contouring accuracy from deep‐learning based tools, and dose‐volume accumulation for patients with multiple treatment sessions.

## 5 CONCLUSION

This study represents the first effort to estimate ABM dose and dose‐volume in a large cohort of radiotherapy patients using electronic medical record data. An automatic, phantom‐based skeletal segmentation tool developed by our group has been used to estimate the dose and dose‐volume metrics within the ABM for a study population of 478 patients treated with photon external beam radiotherapy. Analysis of the resulting dose and dose‐volume estimates indicated that mean dose, the historical exposure variable in epidemiological studies, provides only a limited characterization of the ABM dose distribution. The application of our skeletal segmentation method provided acceptable results for roughly 76% of all tested treatment sessions, with the tool currently underperforming for certain patients due to a range of anatomies and postures that cannot be completely reproduced by phantoms in standardized positions. Ultimately, the dose and dose‐volume estimates from this study population within the MSK‐IMPACT cohort will be connected to tumor and blood sequencing data for use in future analyses on the dose‐response relationship between radiotherapy and clonal hematopoiesis, as well as therapy‐related leukemia. Such future work can provide clarification on the relative impact of different dose and dose‐volume metrics on adverse health effects, which may inform new radiotherapy planning strategies regarding ABM dose and dose‐volume.

## AUTHOR CONTRIBUTIONS

Conception and design of this study was performed by Lindsay M. Morton and Choonsik Lee. Patient data was curated by Lior Braunstein and Kelly L. Bolton. Final analytical study population was selected through quality assurance methods performed by Keith T. Griffin, Kishan J. Pithadia, Yeon Soo Yeom, Lindsay M. Morton, and Choonsik Lee. Image segmentation and active bone marrow dose estimation methods were developed by Keith T. Griffin and Yeon Soo Yeom. Image segmentations were evaluated by Keith T. Griffin and Kishan J. Pithadia. Manuscript was written by Keith T. Griffin and was critically analyzed and revised by all authors. All authors have read and agreed upon the final version of the manuscript.

## CONFLICT OF INTEREST STATEMENT

The authors have no relevant conflicts of interest to disclose.

## Treatment region categorization

In certain analyses of the current work, results have been grouped based on the body region receiving radiotherapy. These groupings were based on body‐region dosimetry methods described in earlier studies from the Childhood Cancer Survivor Study^36^ with treatments classified by eight body regions: the brain, other head, neck, chest, abdomen, pelvis, arm, and leg. Treatment regions from the current work have been classified similarly, with eleven distinct categories. Table A1 provides each treatment region category and example target volumes from this study that correspond to them.

**TABLE A1:** Treatment region categories and example target regions that fall under each category in this study.

Treatment region category	Example target regions
Abdominal	Pancreas, liver, gastroesophageal junction, bile duct
Bone—Extremities	Bone sarcoma (arms, legs)
Bone—Pelvic	Left or right femoral head, sacrum, ilium
Bone—Thoracic	Ribs
Breast	Left or right breast/chest wall, with or without regional lymph nodes
CNS—Intracranial	Frontal lobe, temporal lobe, posterior fossa, whole brain
CNS—Spine	Vertebrae
Extremities	Soft tissue sarcoma (arms, thighs)
Head and neck	Oral cavity (tongue, soft palate), pharynx (tonsils)
Pelvic	Prostate, endometrium, rectum, uterus
Thoracic	Left or right lung, mediastinum, esophagus

Abbreviation: CNS, central nervous system.

## Skeletal alignment comparison and generalizability

Herein we compare the VOF scores produced in the current work against those produced in our pilot study, where ABM dose estimates were previously calculated for 40 patients using both manually and automatically contoured bone sites.^18^ Table A2 summarizes these results. Note that these VOF distributions are not directly comparable. The VOF scores of the current work are for the total patient skeleton on the CT image set and are categorized by treatment region, whereas the VOF results from the prior work are for each manually contoured bone site. Nevertheless, the comparisons that can be made show good agreement. For example, the median VOF for the intracranial treatment region (52.6%) was substantially higher than the cranium (34.4%) and mandible (17.8%) scores of the prior work; the median VOF for treatments to the spine (32.3%) closely matches those results in the vertebrae of the prior work; and median VOF scores for thoracic treatments (breast, bone—thoracic, and thoracic regions) are typically higher than the prior bone‐site specific scores for the clavicles, scapulae, sternum, ribs, and thoracic vertebrae. Results for the bone‐pelvic and pelvic treatment regions also matched reasonably well, considering that the CT image set for such treatments typically also includes the proximal femur, sacrum, and lumbar vertebrae. The average error in the ABM dose estimate was roughly 0.4 Gy in the pilot study, and based on these comparisons, a similar error may be expected in the estimates of the current work. Also note that these comparisons are only for the final analytical study population in the current work; as discussed with Table 2, some skeletal alignments were rejected due to poor performance of the code, and future work will address patient cases where our phantom library does not provide a suitable skeletal surrogate. Those patients excluded based on incomplete information on photon radiotherapy dose (Figure 2) were excluded independent of the dosimetry approach and would not bias our assessment of the method.

Table A3 explores the association between patient characteristics (i.e., age, sex, height, and weight) and the VOF score within the set of accepted segmentations. Calculation of the Spearman's rank‐order correlation coefficient has shown a statistically significant but weak correlation for all four patient characteristics. These findings suggest that our ABM dose estimation method does not preferentially perform worse for certain patient characteristics among adults. As noted in the main text, however, additional testing in a pediatric population is necessary to evaluate the generalizability of this tool to younger patients.

**TABLE A2:** Volume overlap fraction (VOF) distribution metrics from current and prior works.

Distribution of total skeletal VOF (%) from the current work						Mean bone site‐specific VOF (%) from Yeom et al.^18^	
Treatment region	Min.	Q1	Median	Q3	Max.	Bone site	Mean
Abdominal	15.7%	24.0%	26.6%	31.3%	39.9%	Cranium	34.4%
Bone						Mandible	17.8%
*Pelvic*	18.3%	29.4%	39.2%	41.9%	60.3%	Scapulae	21.9%
*Thoracic*	21.7%	26.1%	27.4%	30.3%	39.3%	Clavicles	4.0%
Breast	17.5%	24.6%	26.2%	29.6%	39.3%	Sternum	14.3%
Central nervous system						Ribs	5.1%
*Intracranial*	31.4%	44.9%	52.6%	58.1%	74.9%	Vertebrae‐C	23.2%
*Spine*	16.9%	24.2%	32.3%	38.0%	52.8%	Vertebrae‐T	31.7%
Head and neck	20.8%	29.5%	35.3%	41.5%	51.8%	Vertebrae‐L	37.9%
Pelvic	19.8%	36.8%	44.4%	50.4%	70.3%	Sacrum	36.0%
Thoracic	17.2%	22.4%	25.9%	29.1%	42.8%	Pelvis	51.7%
						Femur‐P	25.6%
						Humerus‐P	14.4%

*Note*: Data for the current work are only shown for the final analytical study population.

**TABLE A3:** Correlations between volume overlap fraction and patient characteristics.

Patient characteristic	Correlation coefficient	*p*‐value
Age	−0.1355	0.0016
Sex	−0.1035	0.0160
Height	0.1457	0.0007
Weight	0.1595	0.0002

*Note*: Non‐parametric spearman correlation was used for this analysis. A *p*‐value < 0.05 was considered significant.
